# Nurses Not in the Lime Light

**Published:** 1916-02-19

**Authors:** M. Neville

**Affiliations:** Children's Hospital, Pendlebury


*To the Editor of* The Hospital.

### Nurses Not in the Lime Light.

*To the Editor of* The Hospital.

Sir,—I should like to say a few words for those nurses who are working hard and trying to keep things up to standard in children's hospitals, without in any way taking from the merit of those nurses who have shown their patriotism by their arduous work in military hospitals. There is no glamour about nursing children now; it is all fire for military work. And, in spite of an ache to be nursing soldiers either at home or abroad, these women feel that they are needed among the children and are doing work for their country.

This branch of work is now more than ever necessary. Excellent work is being done by our nurses, and they are showing a noble spirit, taking many more responsibilities and duties in order that as many of their colleagues as possible may be released for military duty and their posts kept open for them on their return.

Everyone knows how things have become more difficult to work, owing to shortage of labour, delay in deliveries, and many more inconveniences. These nurses are doing all they can to keep things going as usual, training more nurses to draft on to military and civil hospitals. Yet they are unrecognised by the State or by the public eye.

As in the nursing world of to-day there is so much to give and to be gained, these women are some of whom the country may be proud. They are sometimes confronted by the remark that they ought to be in a military hospital nursing soldiers. Yes, but what of our future soldiers? In this time of stress, to an onlooker things seem much the same in a children's hospital—the same amount of work is being done; the same number of probationers trained. Only the spirit of these good women shows that they are taking their share in this war by helping those whom one day they hope will be England's manhood.—I am, Sir, your faithfully,

Children's Hospital, Pendlebury, M. Neville.

